# Inhibition of Notch-1 pathway is involved in rottlerin-induced tumor suppressive function in nasopharyngeal carcinoma cells

**DOI:** 10.18632/oncotarget.19097

**Published:** 2017-07-08

**Authors:** Yingying Hou, Shaoyan Feng, Lixia Wang, Zhe Zhao, Jingna Su, Xuyuan Yin, Nana Zheng, Xiuxia Zhou, Jun Xia, Zhiwei Wang

**Affiliations:** ^1^ The Cyrus Tang Hematology Center and Collaborative Innovation Center of Hematology, Soochow University, Suzhou, China; ^2^ Department of Otolaryngology, Head and Neck Surgery, The Fifth Affiliated Hospital of Sun Yat-sen University, Zhuhai, China; ^3^ Department of Biochemistry and Molecular Biology, Bengbu Medical College, Anhui, China; ^4^ Department of Pathology, Beth Israel Deaconess Medical Center, Harvard Medical School, Boston, MA, USA

**Keywords:** rottlerin, nasopharyngeal carcinoma, Notch-1, invasion, apoptosis

## Abstract

Recent studies have revealed that rottlerin is a natural chemical drug to exert its anti-cancer activity. However, the molecular mechanisms of rottlerin-induced tumor suppressive function have not been fully elucidated. Notch signaling pathway has been characterized to play a crucial role in tumorigenesis. Therefore, regulation of Notch pathway could be beneficial for the treatment of human cancer. The aims of our current study were to explore whether rottlerin could suppress Notch-1 expression, which leads to inhibition of cell proliferation, migration and invasion in nasopharyngeal carcinoma cells. We performed several approaches, such as CTG, Flow cytometry, scratch healing assay, transwell and Western blotting. Our results showed that rottlerin treatment inhibited cell growth, migration and invasion, and triggered apoptosis, and arrested cell cycle to G1 phase. Moreover, the expression of Notch-1 was obvious decreased in nasopharyngeal carcinoma cells after rottlerin treatment. Importantly, overexpression of Notch-1 promoted cell growth and invasion, whereas down-regulation of Notch-1 inhibited cell growth and invasion in nasopharyngeal carcinoma cells. Notably, we found the over-expression of Notch-1 could abrogate the anti-cancer function induced by rottlerin. Strikingly, our study implied that Notch-1 could be a useful target of rottlerin for the prevention and treatment of human nasopharyngeal carcinoma.

## INTRODUCTION

Nasopharyngeal carcinoma (NPC) is an Epstein-Barr virus (EBV)-associated malignant tumor that occurs in nasopharyngeal epithelial tissues. NPC has the highest incidence rate in Southern China and Southeast Asia, which represents a significant disease burden. NPC has an incidence of approximately 2/10,000 per year in endemic areas [1]. Radiotherapy remains the most powerful treatment modality for NPC, especially with the development of advanced imaging and radiation technologies [2]. However, NPC portends a poor prognosis, and the major reason is high rates of recurrence and metastasis. The relapse rate for metastatic patients is as high as 82% by the therapies of combination of radiation and chemotherapy [3]. Therefore, it is required to develop new treatments.

Rottlerin, as a natural plant ployphenol compound, derived from the kalmala tree [4]. Rottlerin has exhibited some biological activities and effects including anti-filarial, anti-bacterial, anti-inflammatory, and immune-regulatory activity [5]. Rottlerin also functions as purgative, anthelmintic, vulnerary, detergent, maturant, carminative, and alexiteric [6]. Recently, Rottlerin possessed anti-cancer property. It is important to note that rottlerin is not the approved drug, although it shows a low toxicity profile in an animal model of Parkinson [7]. Mechanistically, Rottlerin was found to inhibit some protein kinases, such as PKCδ (protein kinase C δ) [8], PRAK (p38-regulated/activated protein kinase), MAPKAP-2 (mitogen-activated protein kinase-activated protein kinase 2), Akt/PKB (protein kinase B), and CaMK (calcium/calmodulin-dependent protein kinase) [9]. Moreover, studies revealed that Rottlerin could induce intrinsic and extrinsic apoptosis by characterization of mitochondria and induction of TRAIL (tumor necrosis factor-related apoptosis-inducing ligand) receptors, DR4 (death receptor 4) and DR5 [10]. Rottlerin could restrain PI3K (phosphatidylinositol 3-kinase)/Akt/mTOR (mammalian target of rapamycin) pathway and active caspase cascade to achieve apoptosis [11].

Notch signaling pathway, an evolutionarily conserved transmission mechanism, plays an important role in various cellular and developmental processes [12]. Notch signaling is implicated in tumor angiogenesis and metastasis [13]. Aberrant activation of Notch signaling has been involved in various malignancies, such as pancreatic cancer, breast cancer, lung cancer and leukemia [14–17]. Notch family includes four members (Notch-1, Notch2, Notch3 and Notch4), and serves as receptors for Notch ligands [18, 19]. Then, upon the binding with Notch ligands, Notch will be cleaved by several proteases, including γ-secretases, to release its intracellular domain (NICD), which can translocate to the nucleus and act as a transcription cofactor to regulate target genes expression in a cell-context-dependent manner [20]. Notch-1 has been shown the oncogenic or tumor suppressive character. Under the condition of hypoxia, Notch-1 could be activated by HIF1α (hypoxia-inducible factor 1-α) in lung adenocarcinoma cells, then the activated Notch-1 could suppress the expression of PTEN (phosphatase and tensin homolog) and activate AKT [21]. The status of p53 has a primary impact on the effects of Notch-1 signaling in lung tumorigenesis [22]. The outcome of targeting Notch-1 in NSCLC (non-small cell lung cancer) patients would be dependent on p53 status [22]. The NF-κB (nuclear factor-kappaB) signaling pathway has been proved to play a critical role in the promotion of proliferation and anti-apoptosis of B-lymphoid tumor cells [23]. One study showed that Notch-1 mutations constitutively activate the NF-κB signaling pathway in CLL (chronic lymphocytic leukemia), and indicating Notch1 and NF-κB could be as potential therapeutic targets in the treatment of CLL [23]. These reports suggest that targeting Notch pathway could be helpful for the treatment of human cancers.

In the present study, we explored the effect of rottlerin in CNE1 and CNE2 cells, such as proliferation, apoptosis, cell cycle, migration and invasion. We also determined whether rottlerin impacted Notch-1 signaling pathway, leading to its anti-cancer properties. We provided evidence that rottlerin inhibited cell proliferation, triggered apoptosis, and arrested cell cycle and retarded cell motility in nasopharyngeal carcinoma cells. More importantly, we identified that rottlerin-induced anti-cancer activity via inhibition of Notch-1 signaling pathway in CNE1 and CNE2 cells. These results elucidated that rottlerin could be a potential efficient agent for the treatment of nasopharyngeal carcinoma.

## RESULTS

### Rottlerin inhibited cell proliferation

**Figure 1:**
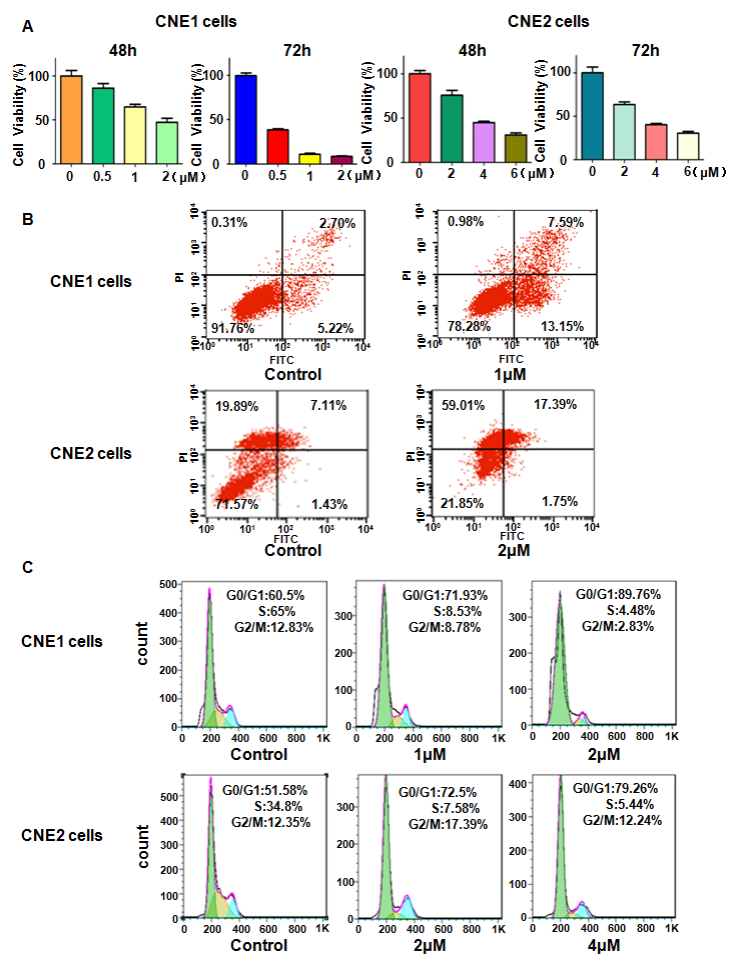
Effect of rottlerin on cell growth, apoptosis, and cell cycle **(A)** Effect of rottlerin on cell growth in nasopharyngeal carcinoma cells was detected by CellTiter-Glo® luminescence assay. Compared to control.Control: DMSO treatment. **(B)** Cell apoptosis in nasopharyngeal carcinoma treated with rottlerin was tested via Flow cytometry. **(C)** Flow cytometry was used to determine cell cycle.

It was proved that rottlerin possessed anti-proliferation activity in human cancer cells. In order to determine whether rottlerin could inhibit cell proliferation in nasopharyngeal carcinoma cells, we measured cell viability by CTG assay in CNE1 and CNE2 cells treated with different concentrations of rottlerin for 48 h and 72 h. Our CTG results showed that cell proliferation was dramatically inhibited by rottlerin in dose-dependent manner (Figure 1A). Moreover, 1 μM and 2 μM rottlerin could inhibit about 30% to 40% of cell proliferation in CNE1 and CNE2 cells after 48 h treatment, respectively (Figure 1A). Therefore, rottlerin significantly inhibited nasopharyngeal carcinoma cells proliferation.

### Rottlerin induced cell apoptosis

Next, we explored whether cell apoptosis was influenced by rottlerin in CNE1 and CNE2 cells. To this end, CNE1 and CNE2 cells were treated with different doses of rottlerin for 48 h, and then apoptotic cells were measured by PI-FITC-annexin assay. Our results showed that cell apoptosis was obviously induced by rottlerin (Figure 1B). Specifically, rottlerin induced cell apoptosis from 7.92 % to 20.74 % in CNE1 cells, from 8.54 % to 19.14 % in CNE2 cells (Figure 1B). Taken together, rottlein stimulated cell apoptosis in nasopharyngeal carcinoma.

### Rottlerin induced cell cycle arrest

We determined whether cell cycle could be arrested in CNE1 and CNE2 cells by rottlerin. Cell cycle analysis was conducted by flow cytometer in CNE1 and CNE2 cells treated with different concentrations of rottlerin for 48 hours. We found that cell cycle was arrested at G1 phase in both NPC cells. CNE1 cells was treated with 1μM and 2μM of rottlerin led to G1 phase from 60.5 % to 71.93 % to 89.76 %, respectively (Figure 1C). Similarly, CNE2 cells were treated with 2μM and 4μM of rottlerin resulted in G1 phase from 51.58 % to 72.5 % to 79.26 %, respectively (Figure 1C). Altogether, rottlerin induced cell cycle arrest at G1 phase in nasopharyngeal carcinoma cells.

### Rottlerin inhibited cell invasion and migration

**Figure 2:**
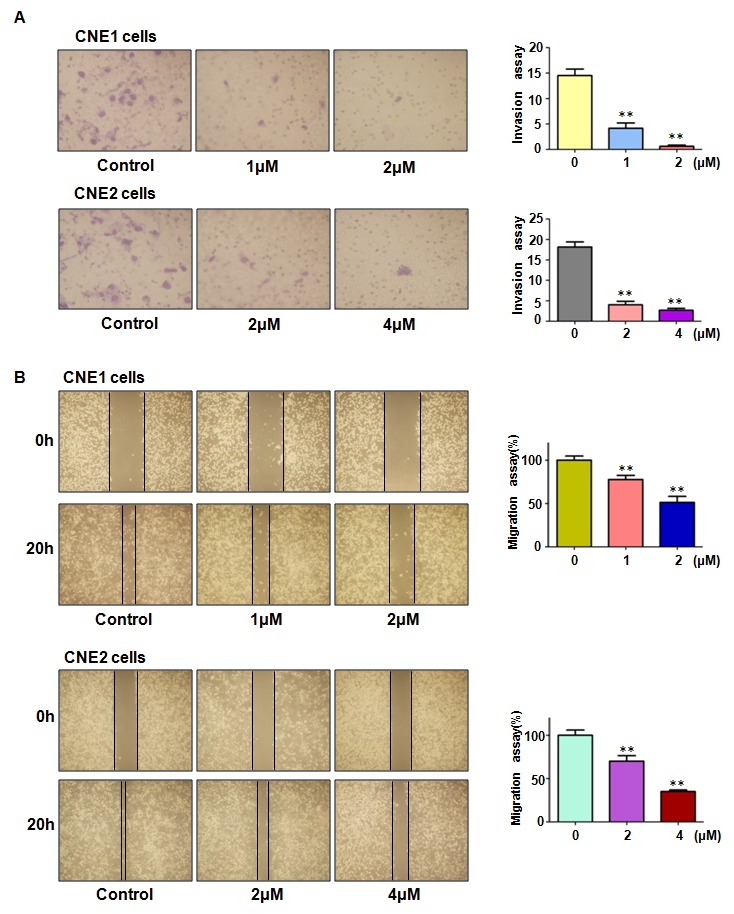
Effect of rottlerin on cell migration and invasion **(A)** Left panel: Rottlerin inhibited cell invasion by Transwell assay in CNE1 cells and CNE2 cells after rottlerin treatment. Right panel: Quantitative results are illustrated for left panel.*P < 0.05, **P<0.01 vs control. **(B)** Left panel: rottlerin inhibited migration of nasopharyngeal carcinoma by wound healing assay. Right panel: Quantitative results are illustrated for left panel. *P < 0.05, **P<0.01 vs control. Control: DMSO treatment.

To explore whether rottlerin controlled cell invasion and migration, we selected Transwell and scratch healing assay to detect this inference. Our transwell assay results showed that rottlerin treatment significantly inhibited cell invasion in dose-dependent manner in nasopharyngeal carcinoma cells (Figure 2A). Our scratch healing assay result demonstrated that the presence of rottlerin decreased the cell migration, and suppressed to more degree with the increase of the dose in CNE1 and CNE2 cells (Figure 2B). These findings indicated that rottlerin inhibited invasion and migration in nasopharyngeal carcinoma cells.

### Rottlerin decreased Notch-1 expression

**Figure 3:**
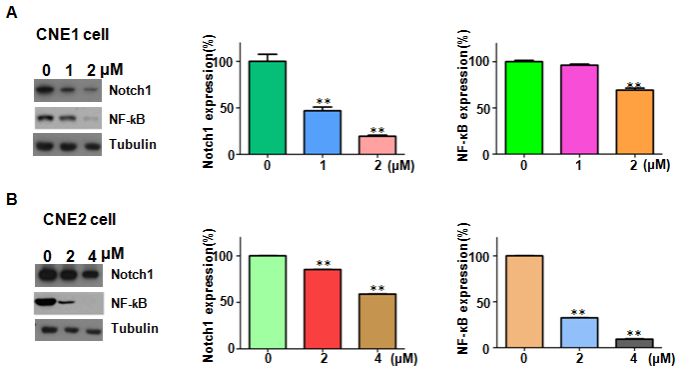
Rottlerin downregulated Notch-1 expression **(A)** Left panel: the expression of Notch-1 and NF-κB was determined by Western blotting in CNE1 cells after rottlerin treatment. Right panel: Quantitative results are illustrated for left panel. *P < 0.05, **P<0.01 vs control.Control: DMSO treatment. **(B)** Left panel: the expression of Notch-1 and NF-κB was determined by Western blotting in CNE2 cells after rottlerin treatment. Right panel: Quantitative results are illustrated for left panel. *P < 0.05, **P<0.01 vs control.

As the above showed, rottlerin exerted its anti-tumor function in nasopharyngeal carcinoma. To future investigated whether rottlerin could decrease Notch-1 expression in NPC cells, we used western blotting to measure the expression of Notch-1 in NPC cells after rottlerin treatment. Our Western blotting results showed that Notch-1 expression was obviously suppressed by rottlerin. Moreover, NF-κB, as a downstream target of Notch-1 [24], was decreased in NPC cells with rottlerin treatment (Figure 3A and 3B). This result suggests that rottlerin down-regulated Notch-1 expression in NPC cells.

### Over-expression of Notch-1 abrogated the anti-proliferation of rottlerin

**Figure 4:**
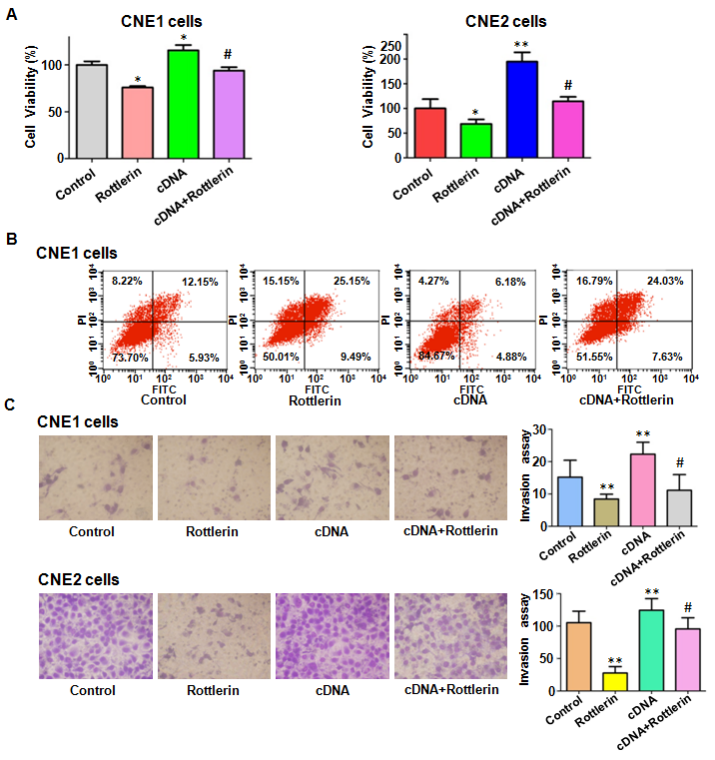
The effect of Notch-1 overexpression on cell growth, apoptosis, and invasion **(A)** CellTiter-Glo® luminescence assay was described the effect of Notch-1 overexpression in combination with rottlerin treatment on nasopharyngeal carcinoma cell proliferation. CNE1: 1μM rottlerin; CNE2: 2μM rottlerin. Control: pcDNA 3.1; cDNA: Notch-1 cDNA; Both: cDNA + rottlerin. *P < 0.05, **P < 0.01, compared with control; ^#^P < 0.05, compared with rottlerin treatment alone or Notch-1 cDNA transfection alone. **(B)** FACS was conducted to investigate cell apoptosis in nasopharyngeal carcinoma cells after Notch-1 cDNA transfection and rottlerin treatment. **(C)** Left panel: Invasion assay was performed in CNE1 and CNE2 cells after Notch-1 cDNA transfection and rottlerin treatment. Right panel: Quantitative results are illustrated for left panel.

In order to validate the role of Notch-1 in NPC, CNE1 and CNE2 cells were transfected with Notch-1 cDNA or empty vector. We found that overexpression of Notch-1 enhanced cell growth in both NPC cells (Figure 4A). Moreover, Notch-1 overexpression abrogated cell growth inhibition by rottlerin treatment in CNE1 and CNE2 cells (Figure 4A). Subsequently, we found that overexpression of Notch-1 significantly inhibited cell apoptosis in NPC cells (Figure 4B). Furthermore, overexpression of Notch-1 abrogated cell apoptosis induced by rottlerin in NPC cells (Figure 4B). These findings suggest that Notch-1 plays a critical role in rottlerin-induced cell growth inhibition and apoptosis.

### Over-expression of Notch-1 rescued the anti-motility of rottlerin

**Figure 5:**
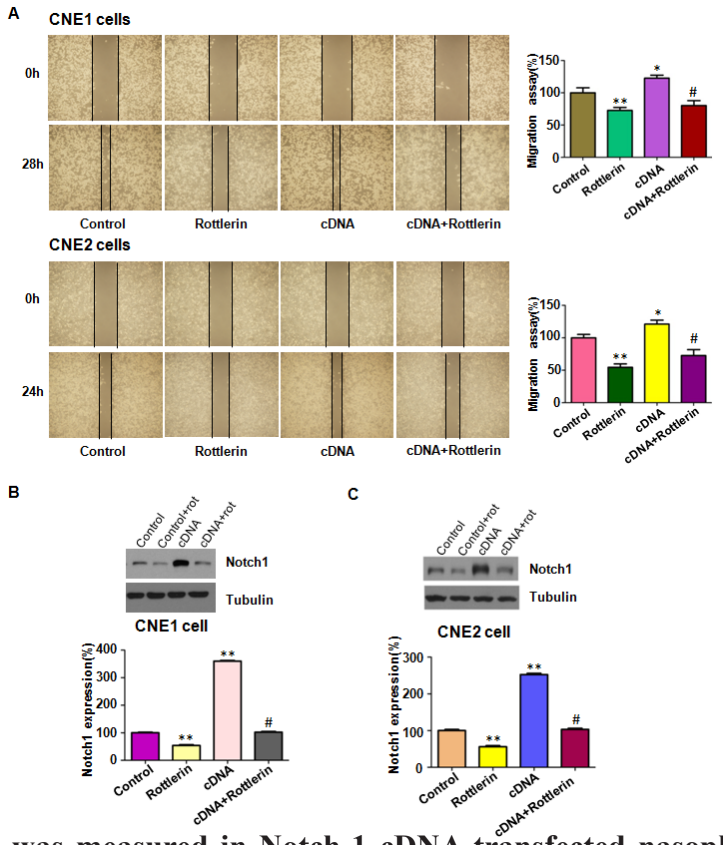
The expression of Notch-1 was measured in Notch-1 cDNA transfected nasopharyngeal carcinoma cells treated with rottlerin **(A)** Left panel: The migration was detected via wound healing assay after Notch-1 cDNA transfection and rottlerin treatment. CNE1: 1μM rottlerin; CNE2: 2μM rottlerin. Control: pcDNA 3.1; cDNA: Notch-1 cDNA; Both: Notch-1 cDNA + rottlerin. Right panel: Quantitative results are illustrated for left panel. *P < 0.05, **P < 0.01, compared with control; ^#^P < 0.05, compared with rottlerin treatment alone or Notch-1 cDNA transfection alone. **(B-C)** Top panel, the expression of Notch-1 was detected by western blotting in nasopharyngeal carcinoma with Notch-1 cDNA transfection and rottlerin treatment in CNE1and CNE2 cells. Bottom panel: Quantitative results are illustrated for left panel. *P < 0.05, **P < 0.01, compared with control; #P < 0.05, compared with rottlerin treatment alone or Notch-1 cDNA transfection alone.

We detected whether Notch-1 overexpression could enhance cell motility in NPC cells in the following study. Our Transwell assay results showed that overexpression of Notch-1 promoted cell invasion in both NPC cell lines (Figure 4C). As we expected, Notch-1 overexpression partly rescued rottlerin-induced cell invasion inhibition (Figure 4C). Moreover, we observed that overexpression of Notch-1 enhanced cell migration in NPC cells by wound healing assay (Figure 5A). Notably, the inhibitory effects of rottlerin on cell migration were attenuated by overexpression of Notch-1 in both NPC cell lines (Figure 5A). We detected the expression of Notch-1 in NPC cells after Notch-1 cDNA transfection in combination with rottlerin treatment. Our results showed that over-expression of Notch-1 abolished the inactivation of Notch-1 by rottlerin (Figure 5B and 5C). These results indicated that rottlerin exerts its antitumor activity partially via inhibition of Notch-1 in NPC cells.

### Down-expression of Notch-1 promoted antitumor activity of rottlerin

**Figure 6:**
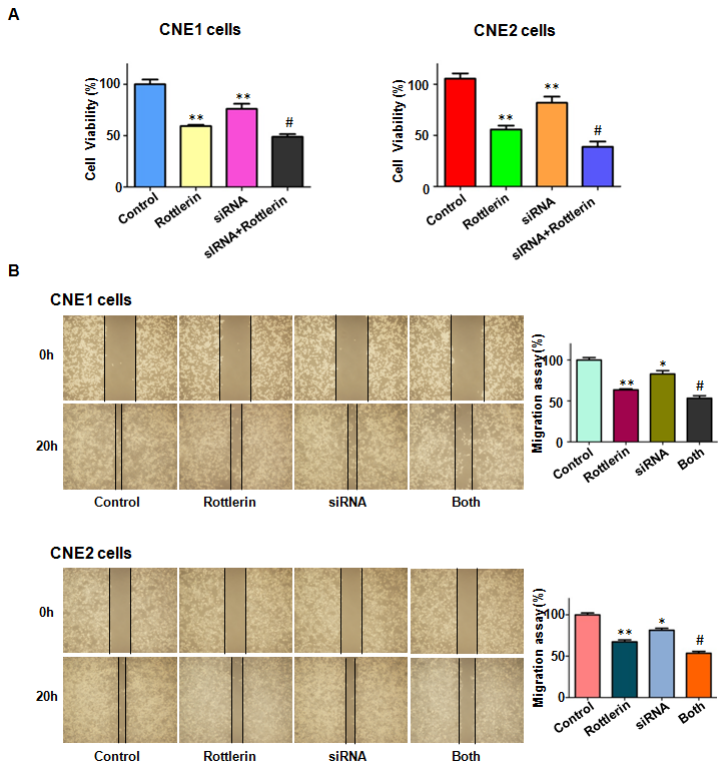
The effect of Notch-1 downregulation on cell growth and migration **(A)** CellTiter-Glo® luminescence assay was used to detect the effect of Notch-1 downexpression in combination with rottlerin treatment on nasopharyngeal carcinoma cell proliferation. CNE1: 1μM rottlerin; CNE2: 2μM rottlerin. Control: siRNA control; siRNA: Notch-1 siRNA; Both: Notch-1 siRNA + rottlerin. *P < 0.05, **P < 0.01, compared with control; #P < 0.05, compared with rottlerin treatment alone or Notch-1 siRNA transfection alone. **(B)** Left panel: The migration was detected via wound healing assay after Notch-1 siRNA transfection and rottlerin treatment. Right panel: Quantitative results are illustrated for left panel. *P < 0.05, **P < 0.01, compared with control; #P < 0.05, compared with rottlerin treatment alone or Notch-1 siRNA transfection alone.

**Figure 7:**
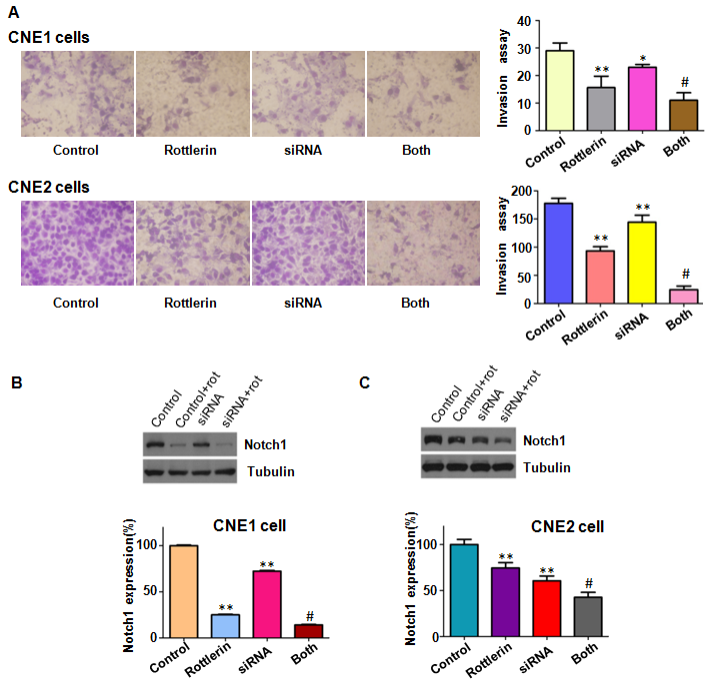
The effect of Notch-1 downregulation on cell invasion **(A)** Left panel: Invasion assay was performed in CNE1 and CNE2 cells after Notch-1 siRNA transfection and rottlerin treatment. Right panel: Quantitative results are illustrated for left panel. CNE1: 1μM rottlerin; CNE2: 2μM rottlerin. Control: siRNA control; siRNA: Notch-1 siRNA; Both: Notch-1 siRNA + rottlerin. *P < 0.05, **P < 0.01, compared with control; #P < 0.05, compared with rottlerin treatment alone or Notch-1 siRNA transfection alone. **(B-C)** Top panel, the expression of Notch-1 was detected by western blotting in nasopharyngeal carcinoma with Notch-1 siRNA transfection and rottlerin treatment. Bottom panel: Quantitative results are illustrated for left panel. *P < 0.05, **P < 0.01, compared with control; #P < 0.05, compared with rottlerin treatment alone or Notch-1 siRNA transfection alone.

To further validate the role of Notch-1 in rottlerin-induced anti-tumor activity, NPC cells were transfected with Notch-1 siRNA in combination with rottlerin treatment. We found that down-regulation of Notch-1 inhibited cell growth in both NPC cells (Figure 6A). Strikingly, down-regulation of Notch-1 enhanced cell growth inhibition induced by rottlerin (Figure 6A). Our wound healing assay results demonstrated that inhibition of Notch-1 retarded cell migration in NPC cells (Figure 6B). Our transwell assay showed that down-regulation of Notch-1 inhibited cell invasion in both NPC cell lines (Figure 7A). Down-regulation of Notch-1 enhanced the inhibitory effects of rottlerin-mediated inhibition of cell migration and invasion in NPC cells (Figure 6B and 7A). Our Western blotting analysis results showed that Notch-1 siRNA transfection enhanced inhibition of Notch-1 induced by rottlerin in NPC cells (Figure 7B).

## DISCUSSION

Recently, multiple studies have reported that rottlerin exerts its anti-cancer activity in human cancers. For example, rottlerin suppressed the expression of LRP6 (low density lipoprotein receptor-related protein-6) and inhibited Wnt/β-catenin and mTORC1 signaling pathway in prostate and breast cancer cells [25]. Lim et al found that rottlerin induced apoptosis through NAG-1 (non-steroidal anti-inflammatory drug activated gene-1) upregulation via ERK (extracellular signal-regulated kinases) and p38 MAPK (mitogen activated protein kinase)-dependent mechanism in colon carcinoma cells [26]. Another studies showed that rottlerin suppressed multiple signaling pathways, leading to induction of apoptosis and cell growth inhibition in pancreatic cancer cells [27]. Rottlerin-induced autophagy leads to the apoptosis in breast cancer stem cells and prostate cancer stem cells [28, 29]. Wang et al. found that rottlerin inhibited cell growth and invasion via down-regulation of Cdc20 (cell divison cycle protein 20) in glioma cells [30]. Yin et al found that rottlerin exerts its anti-tumor activity through inhibition of Skp2 (s-phase kinase associated protein 2) in breast cancer cells and pancreatic cancer cells [31, 32]. Another study indentified that rottlerin down-regulated the expression of TAZ (transcriptional coactivator with PDZ-binding motif) in non-small cell lung cancer [33]. In the current study, we reported that rottlerin suppressed cell growth, arrested cell cycle and induced apoptosis in NPC cells via downregulation of Notch-1 pathway and NF-κB. Interestingly, rottlerin down-regulated NF-κB in a Notch1-independent manner (data not shown).

Accumulated evidences suggested that Notch pathway is involved in carcinogenesis in a variety of human malignancies by regulating many basic cellular processes essential for cancer development and progression. For example, More than 50% of human T-ALLs (T cell acute lymphoblastic leukemia) were caused by activating Notch-1mutations [14]. Notch-1 promoted melanoma progression by inducing β-catenin, which in turn regulated cyclin D1 expression [34]. Additionally, breast cancer patients with Notch-1 positive had shorter disease-free survival, indicating that Notch-1 may be involved in metastasis and is closely correlated with breast cancer stem cells [35]. Downregulation of Notch-1 by expressing its short hairpin RNAs (shRNAs) in Notch-1-positive NSCLC (non small cell lung cancer) cell lines significantly inhibited their anchorage independent growth in soft agar [36]. Chen et al found that suppression of Notch signaling pathway by γ-secretase inhibitor inhibited nasopharyngeal carcinoma cell proliferation [37]. Furthermore, down-regulation of Notch signaling pathway by γ-secretase inhibitor enhanced the radiosensitivity of nasopharyngeal carcinoma cells [38]. Moreover, inhibition of Notch3 signaling pathway significantly enhanced sensitivity to cisplatin in EBV-associated nasopharyngeal carcinoma [39]. Strikingly, Notch inhibition suppressed nasopharyngeal carcinoma by depleting cancer stem-like side population cells [40]. Zhou et al. reported that GSI combined with cisplatin enhanced apoptosis of nasopharyngeal carcinoma cells [41]. In line of these reports, we found that downregulation of Notch-1 inhibited cell growth and invasion, while overexpression of Notch-1 enhanced cell proliferation and invasion.

Interestingly, Notch signaling pathway was validated to play tumor suppressive functions in several types of human cancers, suggesting that Notch functions depend on the cellular context [42]. Since Notch-1 could be an effective target to treat nasopharyngeal carcinoma, development of Notch-1 inhibitors is important for the treatment of NPC. Several GSIs have been developed for clinical trials. However, GSIs exhibits non-specific inhibition of Notch receptors and multiple other γ-secretase substrates [43]. GSIs are associated with cytotoxicity in the gastrointestinal tract [43]. Natural compounds have been identified as the inhibitors of Notch signaling pathway. Curcumin was reported to inhibit the DNA-binding ability of ICN and suppress the MT1-MMP and MMP2 proteins in prostate cancer cells [44]. Genistein inhibited Notch-1 expression in pancreatic cancer cells [45]. One study showed that corilagin suppressed cholangiocarcinoma progression through Notch signaling pathway *in vitro* and *in vivo* [46]. Another study revealed that alpinetin targets glioma stem cells by suppression of Notch pathway [47]. Our study suggests that rottlerin could be a new inhibitor of Notch-1 in nasopharyngeal carcinoma. More experiments are necessary to determine how rottelrin inhibited Notch-1 expression. Does rottlerin inhibit γ-secretase in NPC cells? Does rottlerin regulate the upstream genes of Notch-1? Does rottlerin govern miRNAs that target Notch-1 expression? Does rottlerin upregulate Fbw7 that degrades Notch-1 level? Further investigation is required to explore whether rottlerin exerts its anti-tumor activity via inhibition of Notch-1 in animal mouse model.

## MATERIALS AND METHODS

### Cell culture, reagents and antibodies

Human nasopharyngeal carcinoma CNE1 and CNE2 cells were cultured in DMEM medium with 10% fetal bovine serum and 1% penicillin and streptomycin in a 5% CO_2_ at 37°C. Primary antibody for Notch-1 (recognize ICN, sc-6014, 1:1000) was purchased from Santa Cruz Biotechnology (Santa Cruz, CA). And NF-κB p65 (#9936, 1:1000) antibody was brought from Cell Signaling Technology. Then monoclonal Anti-Tubulin was purchased from Sigma-Aldrich (St. Louis, MO). All secondary antibodies were purchased from Thermo Scientific. Rottlerin (CAS number 82-08-6, 85% rottlerin) was obtained from Sigma-Aldrich (St. Louis, MO). Rottlerin was dissolved in DMSO to make a 10 mM stock solution and was added directly to the medium at different concentrations. Lipofectamine 2000 was purchased from Invitrogen. CellTiter-Glo (®) luminescent cell viability assay was purchased from Promega (Madison, WI). Cells were treated with 0.1% DMSO as the control group.

### Cell viability assay

Cells were seeded into 96-well plates (5×10^3^ cells/well) for overnight incubation and then treated with different concentrations of rottlerin. After 48 h and 72 h treatment, cell viability was assessed using the CellTiter-Glo® luminescence (CTG) assay. Each value was normalized to cells treated with DMSO.

### Cell apoptosis assay

Exponentially growing cells (3 × 10^5^ cells/well) were cultured in a six-well plate overnight and treated with various concentrations of rottlerin for 48 h. After trypsinizion the cells were washed with PBS, then resuspended in 500 μl binding buffer with 5μl Propidium iodide (PI) and 5μl FITC-conjugated anti-Annexin V antibody. Apoptosis was analyzed by a FACScalibur flow cytometer (BD, San Jose, CA, USA)[48].

### Cell cycle analysis

Cells (3× 10^5^ cells/well) were seeded in a 6-well plate overnight and then treated with 1 μM and 2 μM rottlerin for 48 h. After 48 h, cells were collected and washed with PBS. Then, suspended cells with 70% cold alcohol were kept at 4°C overnight. Prior to analysis, the cells were washed with cold PBS, and re-suspended at 1×10^6^ cells/ml in PBS. Cells were incubated with 0.1 mg/ml RNase I and 50 mg/ml Propidium iodide (PI) for 30 min. Cell cycle was analyzed with a FACScalibur flow cytometer (BD, San Jose, CA).

### Cell wound healing assays

CNE1 and CNE2 cells were cultured in 6-well plates. After cells converged almost 100%, scratched the cells with a 200 μl yellow pipette tips, and then absorbed the supermatant cells washed with PBS. Treated with different concentrations of rottlerin to cells and incubated for 20h. The scratched area was photographed with a microscope at 0 h and 20 h, respectively [49].

### Transwell invasion assay

The transwell invasion assay was performed using a 24-well plate with 8-mm pore size chamber inserts (corning, New York, NY, USA) and Matrigel (BD Biosciences). The cells were treated with rottlerin or Notch-1 transfection or combination, and seeded into the upper chamber of insert, which were suspended in serum-free culture medium. Then, complete medium was added into the under chamber. After incubation for 24 h, the invaded cells in the membrane of the chamber were stained with Wright’s-Giemsa, and then photographed with a microscope.

### Transfection

CNE1 and CNE2 were transfected with Notch-1 cDNA or Notch-1 siRNA or empty vector using lipofectamine 2000 following the manufacture’s instruction. Notch-1 siRNA sense: 5’-CCG UCA UCA AUG GCU GCA ATT-3’; antisense: 5’UUG CAG CCA UUG AUG ACG GTT-3’. They were purchased from GenePharma (Shanghai, China). After the transfection about 2 days, these cells were harvested to future analysis as a part of the results.

### Western blotting analysis

Cells total protein were extracted, and then tested protein concentrations by BCA Protein Assay kit (Thermo Scientific, MA). Each containing 40 μg of total protein were separated by 10% SDS-polyacrylamide gel and transferred to Polyvinylidene Flouride (PVDF) membranes. Then the membranes were blocked by 5% nonfat milk in TBST at room temperature. The immunoblots were probed with primary antibodies overnight at 4 °C. After washed with TBST for three times and then incubated with secondary antibody for 1h at room temperature. The immune reaction was visualized detected by electrochemiluminescence (ECL) assay [50].

### Statistical analysis

All date were analyzed using GraphPad Prism 4.0 (Graph Pad Software, La Jolla, CA). Student’s *t-test* was used to evaluate statistical significance with a threshold of P values less than 0.05 in three groups. ANOVA was performed to evaluate statistical differences in four groups.
